# CARING FOR CHILDREN WITH DISABILITIES, WORKING, AND SAVING FOR RETIREMENT OVER THE LIFE COURSE

**DOI:** 10.1093/geroni/igac059.2907

**Published:** 2022-12-20

**Authors:** Shirley Porterfield, Eunsun Kwon

**Affiliations:** University of Missouri-St. Louis, Saint Louis, Missouri, United States; Fairleigh Dickinson University, Florham Park, New Jersey, United States

## Abstract

In the US, financial security in retirement depends on having savings and other assets accumulated during the pre-retirement years. Pension savings accrue over the working life, in defined-benefit or defined-contribution retirement plans. Mothers with children with disabilities experience different life-course work trajectories than mothers without children with disabilities, though data used in previous studies primarily examine a single point in time or a short span of years. This paper draws longitudinal data from the nationally-representative 1979 National Longitudinal Survey of Youth for the years 1987 through 2018, using a life-course perspective and sequence analysis to identify long-term work patterns among women with children who do and do not have disabilities with an explicit focus on variations in occupational class and employment status. We found a distinctive pattern of long-term work history with five types: Full-time semi-professional to not working, Constantly not working, Semi-professional full time, Professional full time, and Not working to full time work. Results from regression analyses revealed variation in mothers’ household financial preparation for retirement at late mid-adulthood. Compared with mothers who held professional full-time jobs throughout their adulthood, mothers who started full time jobs in middle age and have children with disabilities were less likely to have pension plans. Mothers of children with disabilities who left the labor force in early middle age tended to have lower retirement savings. Policy interventions to address these mothers’ caregiving ability to stay engaged in the workforce and prepare for their retirement need to be explored.

